# Therapeutic Potential of Dehydroepiandrosterone in Reversing Age‐Related Neurodegenerative and Cognitive Declines in Rats

**DOI:** 10.1002/alz70855_102078

**Published:** 2025-12-23

**Authors:** Pardeep Kumar

**Affiliations:** ^1^ F H Medical College & Hospital, Agra, Uttar Pradesh, India

## Abstract

**Background:**

Dehydroepiandrosterone (DHEA), a neurosteroid synthesized in the brain, declines significantly with age, making neurons more vulnerable to neuromodulatory changes. This study aimed to evaluate the neuroprotective potential of DHEA by analyzing its effects on acetylcholinesterase (ACh), tyrosine hydroxylase (TyrH), monoamine oxidase B (MAO‐B), glucose transporter 4 (GLUT4) expression, membrane fluidity, neurolipofuscin, superoxide dismutase (SOD) activity, and cognitive performance. Behavioral tests and molecular analyses were performed in male rats of three age groups: young (4 months), adult (14 months), and old (24 months). The study also investigated whether DHEA administration could restore these parameters to youthful levels.

**Methods:**

Aged rats (14 and 24 months old) were administered daily subcutaneous injections of DHEA (30 mg/kg body weight) for one month. After treatment, animals were sacrificed, and synaptosomes were isolated for biochemical and molecular analyses. Cognitive performance was assessed using the Morris water maze, while ultrastructural changes in brain regions were evaluated through MRI.

**Results:**

Aging was associated with significant increases in MAO‐B activity, lipid peroxidation, and neurolipofuscin accumulation, alongside decreases in SOD activity, TyrH and ACh levels, membrane polarization, and GLUT4 expression. DHEA treatment significantly improved cognitive performance and memory in aging rats and elevated synaptic proteins, such as synaptophysin and synapsin I. Ultrastructural examination of the frontal cortex revealed reductions in lipofuscin and lysosomal degradation following DHEA administration. Biochemical parameters also returned to near‐normal levels in aged rats treated with DHEA.

**Conclusions:**

This study highlights the therapeutic potential of DHEA as a neuroprotective and anti‐aging agent. DHEA effectively mitigates age‐related molecular, behavioral, and biochemical changes, suggesting its role as a promising adjunct therapy for addressing age‐associated neuronal decline. Further research is warranted to explore its clinical implications.

